# Synergistic effects of micro- and macro-sized palm kernel shell fillers on the tensile properties of HDPE composites

**DOI:** 10.1098/rsos.241911

**Published:** 2025-07-23

**Authors:** Abdul-Manan Kayaba, Obed Issakah, Stefania Akromah, E. E. Nettey-Oppong, Eric Kwame Anokye Asare

**Affiliations:** ^1^ Department of Materials Engineering, Kwame Nkrumah University of Science and Technology, Kumasi, Ghana; ^2^ KNUST Center for Engineering Materials Research (KCEMR), Kwame Nkrumah University of Science and Technology College of Engineering, Kumasi, Ashanti Region, Ghana

**Keywords:** high-density polyethylene, palm kernel shells, particulate composites

## Abstract

In this study, high-density polyethylene (HDPE) composites reinforced with palm kernel shell (PKS) fillers with mixed particle sizes were prepared using melt-extrusion compounding. A 5-ton hydraulic hot-press machine was employed to fabricate samples for tensile testing, with a focus on understanding the influence of varying filler sizes on the mechanical properties of the HDPE/PKS composite. The 30 wt% PKS composites demonstrated an elastic modulus (E) of 1.08GPa, ultimate tensile strength (UTS) at 14.13MPa, yield strength at 8.6MPa, stress at failure 12.87MPa, and elongation at failure 5.16%. However, the incorporation of larger PKS particles (PKSL) had a detrimental effect on the tensile properties, with increasing PKSL content leading to significant reductions in tensile properties. For example, for 7.5 wt% PKSL, E decreased by approximately 18%, yield strength by 37%, UTS by 24%, stress at failure by 29%, and total elongation by 62%. Similar trends were observed for the composites containing 15 wt% and 22.5 wt% PKSL. Differential scanning calorimetry (DSC) and thermogravimetric analysis (TGA) were employed to assess the melting temperature ranges and thermal stability of the composites, respectively. Scanning electron microscopy (SEM) provided insights into the failure mechanisms, revealing weak filler-matrix interfacial bonding with larger particles, resulting in debonding and ultimately compromising the tensile properties of the composite.

## 1. Introduction

High-density polyethylene (HDPE) is a widely used thermoplastic polymer known for its impressive mechanical properties, including tensile strength (ranging from 7.6 to 43.0 MPa), rigidity, impact resistance, strength-to-weight ratio (8.17–45.26 kPa/(kg m^˗3^)), stability at room temperature and excellent chemical resistance. With the melting temperature of 120℃–130℃, HDPE’s versatility makes it suitable for a wide range of applications, including packaging, electronics, construction and marine industries [1,2]. Despite its broad applicability, HDPE has inherent limitations in terms of its tensile strength, impact resistance and hardness. These drawbacks limit their performance in applications that require high mechanical durability, such as manufacturing equipment and automotive components. To address these limitations, researchers have focused on incorporating fillers, both bio-based and synthetic, into the HDPE matrix to enhance its mechanical properties [3,4]. In particular, bio-based fillers have garnered significant attention owing to their abundance, cost-effectiveness, lightweight nature and environmental sustainability [5]. The addition of natural fillers, such as kenaf, hemp, sisal and ground coffee, to HDPE has demonstrated improvements in properties such as tensile strength, impact resistance and hardness [6].

Numerous studies have highlighted the benefits of using bio-based fillers in polymer matrices. For instance, the addition of coffee ground fillers to HDPE has been shown to increase the tensile strength by up to 25%, tensile modulus by up to 24% and impact strength by up to 6% [7]. Coconut shells, when used as fillers in polymers such as low-density polyethylene (LDPE), improve hardness, but may compromise tensile strength, impact resistance and ductility as filler content increases [8]. However, the hydrophilic nature of these fillers poses a challenge by weakening the interfacial bonding between the filler and the polymer matrix, which can negatively affect the tensile, flexural and hardness properties of the composites [9,10].

Palm kernel shell (PKS), a byproduct of the palm oil industry, is abundant in Africa, with Ghana alone producing approximately 2.5 million tons annually [11]. PKS is recognized for its high strength and hardness, which are largely attributed to its significant lignin content, typically ranging from 44% to 50.7% [12]. In addition to its mechanical robustness, PKS also offers additional advantages as a bio-based filler, including its lightweight nature and good thermal stability, making it a promising candidate for composite material reinforcement [13,14]. Research on the use of PKS as a filler in composite materials has demonstrated its potential to enhance mechanical properties. For instance, the incorporation of PKS fillers into a natural rubber matrix has been shown to improve tensile strength, modulus, and elongation at fracture [10]. Similarly, when 10−15 wt% of PKS particles with sizes ranging from 150 to 212 µm were added to HDPE, there were significant improvements in the elongation at break and modulus of elasticity, increasing by 40%–80% and from 1095 to 1150 MPa, respectively [15].

Herein, we investigated the effect of mixed PKS particle size formulations on the tensile properties of HDPE/PKS composites. It is well established that smaller particles tend to enhance the tensile strength, whereas larger particles may have the opposite effect. Therefore, this study seeks to determine whether there is an optimal composition of mixed particle sizes that can maximize the tensile properties of the composite. The morphological characteristics of the composite samples were analysed using optical microscopy (OM) and scanning electron microscopy (SEM). Thermal characterization was performed using simultaneous thermal analysis (STA), and the tensile properties were assessed through standard quasi-static tensile tests. This comprehensive study aims to advance the understanding of how mixed PKS particle sizes affect both the tensile properties and the thermal behaviour of HDPE composites.

## 2. Material and methods

### 2.1. PKS particle preparation

PKS residue was obtained from a local palm oil processing company in Ayeduase, located in the Kumasi metropolitan area of Ghana. PKS was crushed and milled using a corn milling machine. The crushed material was thoroughly washed with water and air-dried for 24 h. Once dried, the PKS was sieved to separate the particles into two distinct size fractions: 1 mm and 250 μm.

### 2.2. PKS/HDPE composite preparation

The composite samples were prepared with a fixed HDPE pellet (Sabic®) at 70 wt% while varying the ratios of 250 μm (PKS_S_) and 1 mm (PKS_L_) particles, as indicated in table 1. The mixture was pelletized twice using a Rigidex HD5218EA mini extruder, which operated at a screw speed of 50 rpm and a temperature of 200°C for 5 min. The resulting pellets were then placed in a mould and subjected to hot pressing at 200°C using a 5-ton CNCESTTM hydraulic press to form the final composite samples.

**Table 1.** Preparation of mixed filler size composite in various weight percentages.

HDPE (wt%)	PKS_S_ (wt%)	PKS_L_ (wt%)
70	30	0
70	22.5	7.5
70	15	15

### 2.3. Optical and scanning electron microscopy

The particle size distribution of the PKS particles was characterized using OM. Optical micrographs were captured using an optical microscope, and the particle size distribution was analysed using ImageJ software (version 1.8.0). The morphological features of the fractured surfaces of the tensile test specimens were examined using SEM. SEM analysis was conducted using a JEOL JSM-IT300 microscope, and all specimens were sputter-coated with silver prior to imaging to ensure adequate conductivity for analysis.

### 2.4. Tensile properties

The tensile properties of PKS-reinforced HDPE composites were evaluated using dog-bone specimens with a gauge length of 15 mm. The average thicknesses and weights of the specimens were recorded and used to calculate their mechanical properties. Quasi-static tensile tests were performed using a Shimadzu testing system equipped with a 1 kN load cell. To ensure secure gripping during testing, the clamping ends of the dog-bone specimens were sandwiched between two pieces of sandpaper before being fixed to the grips of the testing system. The specimens were subjected to uniaxial tensile loading until failure with a crosshead displacement rate of 1 mm/min. The axial deformation was measured using a non-contact video gauge extensometer. Two dots were marked on each specimen and the gauge length was defined as the distance between the dots. The strain was calculated as the ratio of the change in gauge length to the original gauge length.

### 2.5. Simultaneous thermal analysis

Simultaneous thermal analysis (STA) was employed to investigate the thermal characteristics of the composite specimens using an SDS QSERIES device. The analysis was performed over the temperature range of 40°C–800°C at a heating rate of 10°C/min. Thermogravimetric analysis (TGA) and derivative thermogravimetry (DTG) were used to assess the thermal degradation stability and estimate the associated thermal properties, including the onset and peak degradation temperatures. Differential scanning calorimetry (DSC) was used to determine the melting temperature range of the composite specimens.

### 2.6. Fourier transform infrared radiation (FTIR)

FTIR was employed to elucidate the chemical composition of the PKS. The PerkinElmer ATR-FTIR device was used. The spectra were generated over a wavenumber range of 500 and 4000 cm^-1^.

## 3. Results and discussion

### 3.1. Chemical composition

**Figure 1.**
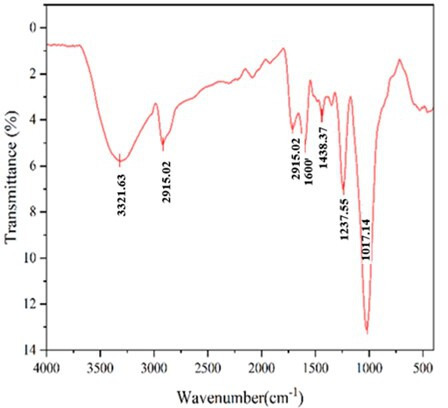
FTIR spectra of palm kernel shell (PKS). FTIR spectra of PKS illustrating key absorption peaks: O–H stretching at 3321.63 cm⁻¹, C-H stretching at 2915.02 cm⁻¹, C=C stretching at 1600 cm⁻¹, C=O stretching at 1710.20 cm⁻¹, and C–O stretch vibrations of C–H and stretching vibrations of C–O at 107.42 cm⁻¹, indicating the presence of lignin and carbohydrate groups.

FTIR spectroscopy analysis of PKS revealed distinct peaks corresponding to various chemical components, as illustrated in figure 1. The presence of methyl groups from lignin, cellulose and hemicellulose is indicated by the O–H stretching bond at 3321.63 cm⁻¹ and the C–H stretching bond at 2915.02 cm⁻¹ [16]. The aromatic component of hemicellulose is represented by the C = C stretching peak at 1600 cm⁻¹ [17]. The peak at 1710.20 cm⁻¹ corresponds to the C = O stretching of carbonyl functional groups, which are attributed to lignin and hemicellulose [18]. Additionally, the peak at 1237.55 cm⁻¹, falling between 1500 and 1000 cm⁻¹, corresponds to the C–O stretch vibration of ether, aryl, and alkyl groups within the lignin [19]. The peak observed at 1017.42 cm⁻¹, extending to 600 cm⁻¹, is indicative of C–H bending vibrations and C–O stretching vibrations, further supporting the presence of lignin and carbohydrate groups.

### 3.2. Particle size distribution

**Figure 2.**
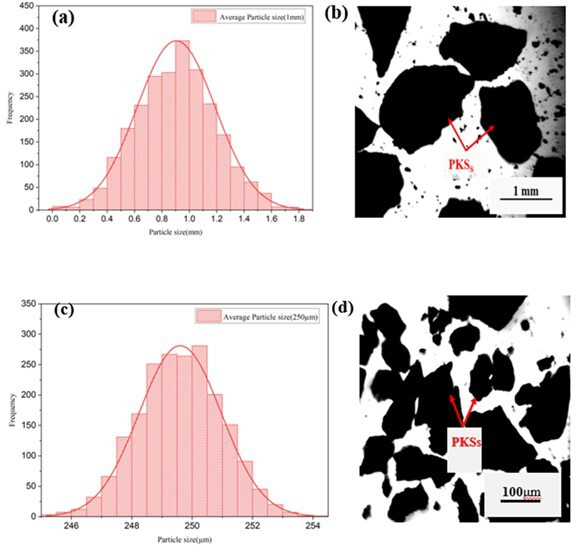
Optical micrographs and particle size distribution of PKS. Optical micrographs showing the different particle sizes of PKS used in the composites. The accompanying normal distribution curves illustrate the size distribution of the smaller (250 μm) and larger (1 mm) PKS particles.

The optical micrographs and normal distribution curves presented in figure 2 provide insight into the particle size distribution of the PKS used in the composites. The analysis revealed that the average particle sizes of the smaller and larger PKS particles were 250 µm and 1 mm, respectively. These size distributions are crucial for understanding the reinforcement characteristics of PKS in the HDPE matrix.

### 3.3. Surface morphology

**Figure 3.**
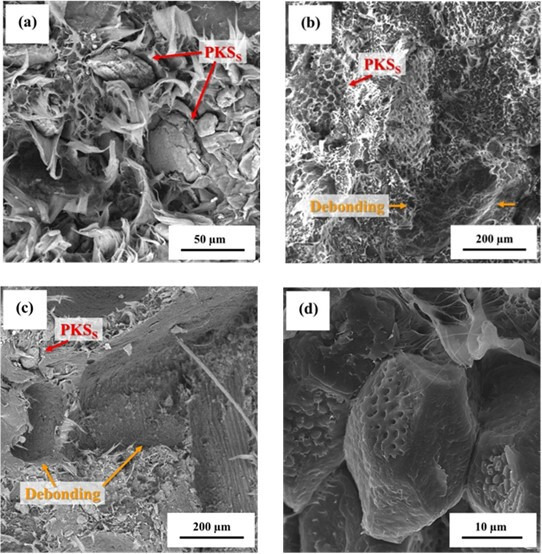
Surface morphology of fractured PKS/HDPE composites. SEM images of the fracture surfaces of PKS/HDPE composite samples. Panel (a) displays typical craze fibrils and dimples indicative of ductile failure. Panels (b) and (c) show prominent debonding sites in samples with 22.5 wt% and 15 wt% PKS particles, respectively. Panel (d) illustrates craze fibrils and dimples on PKS particle surfaces post-failure, suggesting significant debonding as a key failure mechanism.

The surface morphologies of the fractured samples are shown in figure 3. The fracture surfaces exhibited features such as craze fibrils and dimples, which are characteristic of ductile failure in HDPE specimens subjected to uniaxial loading at low strain rates [20]. Notably, the samples with 22.5 wt% PKS (figure 3b) and 15 wt% PKS (figure 3c) displayed significant debonding sites. The presence of craze fibrils and dimples in these areas, along with the observation of similar features on the surfaces of the PKS particles post-failure (figure 3d), strongly suggests that debonding between the PKS particles and HDPE matrix was a major contributor to the failure mechanism. This debonding likely compromised the structural integrity of the composite, leading to an observed reduction in the tensile properties.

### 3.4. Tensile properties

**Figure 4.**
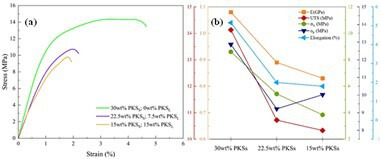
Tensile properties of PKS/HDPE composites. (a) Stress–strain curves for HDPE composites containing 30 wt% PKS, showing quasi-linear deformation up to yield, followed by plastic deformation. Average tensile properties are reported: Young’s Modulus (E) = 1.08 GPa, Yield Strength (σY) = 8.6 MPa, ultimate tensile strength (UTS) = 14.1 MPa, Stress at Failure (σF) = 12.9 MPa, and Total Elongation (εF) = 5.2%. (b) Comparative tensile properties of composites with varying contents of largersized PKS particles (7.5 wt% and 15 wt%), illustrating reductions in E, σY, UTS, σF and εF. Notable decreases are observed with increased PKS particle size.

Figure 4a illustrates the typical stress–strain curves of the tested PKS/HDPE composite samples along with their respective tensile properties. The stress–strain behaviour of all specimens initially exhibited a quasi-linear deformation up to approximately 1% strain, beyond which yielding began. The samples containing 30 wt% PKS particles displayed a distinct yield point and substantial plastic deformation prior to failure. The average tensile properties for these samples are as follows: Young’s Modulus (*e*) = 1.08 GPa; Yield Strength (σY) = 8.6 MPa; ultimate tensile strength (UTS) = 14.1 MPa; stress at failure (σF) = 12.9 MPa; and total elongation (εF) = 5.2%.

In contrast, the composites incorporating larger PKS particles exhibited reduced tensile properties, as shown in figure 4b. For samples with 7.5 wt% large-sized PKS (PKSL), the elastic modulus decreases to 0.9 GPa, reflecting an approximately 18% reduction. Yield Strength drops to 5.4 MPa (a 37% reduction), UTS to 10.7 MPa (24% reduction), stress at failure to 9.2 MPa (29% reduction), and total elongation to 2% (a 62% reduction). These reductions were even more pronounced with an increase in 15 wt% PKSL. Notably, the elastic moduli of these composites exhibited minimal sensitivity to variations in particle size, which is consistent with observations in the literature that micron-sized fillers have a limited impact on the elastic modulus [21]. Table 2 provides an overview of the tensile properties measured for the PKS/HDPE composite samples, including the Young’s modulus, yield strength, UTS, stress at failure, and total elongation. Values are reported for different formulations of PKS particles and their respective sizes.

**Table 2.** Summary of the measured tensile properties.

tensile properties	30 wt% PKS_S_	22.5 wt% PKS_S_	15 wt% PKS_S_
E (GPa)	1.08 ± 0.04	0.89 ± 0.17	0.83 ± 0.09
UTS (MPa)	14.13 ± 0.76	10.72 ± 1.05	10.33 ± 0.55
yield strength (MPa)	8.6 ± 0.95	5.42 ± 1.07	3.84 ± 0.23
stress at failure (MPa)	12.87 ± 0.83	9.22 ± 1.18	10.02 ± 0.94
elongation at failure (%)	5.16 ± 0.59	2.0 ± 0.25	1.8 ± 0.11

The observed trends align with established findings that the tensile properties of particulate-polymer composites generally decrease with increasing filler particle size. Smaller particles typically result in a higher tensile strength because of the greater surface area available for filler-matrix bonding, which enhances the interfacial bond strength and bond density throughout the composite [22]. Finer grinding of PKS particles likely leads to partial defibrillation of lignocelluloses, improving the accessibility of hydroxyl groups associated with cellulose fibrils for bonding with the matrix [23].

Failure in composite materials often originates from one or more of the following phenomena: fracture of filler particles, formation of microcracks within the matrix or debonding between the filler and matrix [24]. In composites with natural fillers, debonding is the most prevalent failure mode, attributed to weak interfaces between the fibres and matrix, and low bond density resulting from chemical incompatibility [25]. The stress required to initiate interfacial debonding is directly related to the filler-matrix interfacial bond strength and bond density. Composites with a higher proportion of smaller particles generally exhibit higher interfacial bond strength and bond density, thereby increasing toughness owing to lower strain energy release rates [21,24,26]. Furthermore, debonding in composites with smaller particles (<200 µm) tends to initiate at approximately 70% of the failure stress, whereas for larger particles, debonding begins at>90% of the failure stress, leading to an earlier onset of failure [21].

### 3.5. Thermal properties

**Figure 5.**
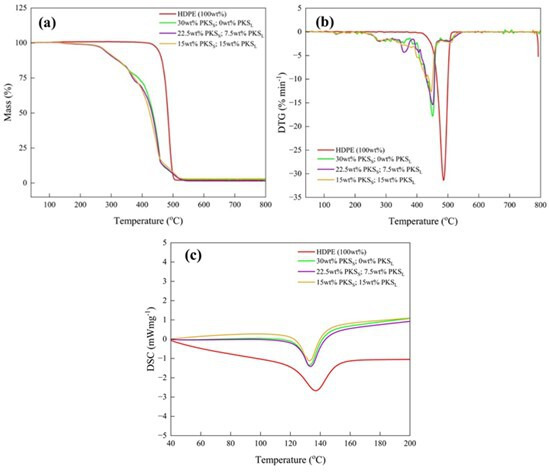
Thermal properties of PKS/HDPE composites. (a) TGA curves showing the degradation profiles of neat HDPE and PKS/HDPE composites. The onset decomposition temperature of HDPE decreases significantly with PKS addition, from ~380 °C to ~255 °C, and the peak decomposition temperature shifts from ~480 °C to ~450 °C. (b) DTG curves revealing multiple peaks corresponding to different thermal degradation stages: hemicellulose at 275 °C, cellulose at 355 °C, and lignin at 500°C. (c) DSC curves indicating the melting temperature of neat HDPE (~137 °C) and its reduction to 132°C–134 °C with the incorporation of PKS particles, with no significant correlation to particle size ratios.

Figure 5 presents the thermal analysis results, including the TGA, DTG and DSC profiles for the PKS/HDPE composites. The thermal analysis did not reveal substantial changes in the thermal properties compared to previous findings [27]. The TGA graph (figure 5a) shows a single-step degradation curve for pure HDPE, with thermal degradation occurring between 420°C and 500°C. The corresponding DTG curve features a single peak at approximately 480°C, which aligns with the decomposition temperature of HDPE [28]. However, the addition of PKS particles significantly affected the thermal stability of the HDPE. Specifically, the onset temperature of decomposition of the composites decreased from approximately 380°C for neat HDPE to approximately 255°C. Similarly, the peak decomposition temperature shifted from approximately 480°C to approximately 450°C. These changes are consistent with observations of polymer composites reinforced with natural fillers [29].

The TGA curves for the PKS/HDPE composites revealed a multistep degradation process, indicative of the presence of PKS. The DTG profiles exhibited multiple peaks between 200°C and 600°C, excluding the HDPE decomposition peak, which is characteristic of PKS [30]. The peak at 275°C corresponds to the thermal depolymerization of hemicellulose, which typically occurs between 225°C and 325°C [31]. The peak at 355°C is attributed to cellulose degradation, which generally occurs between 300°C and 400°C [32]. Additionally, the peak at 500°C is likely associated with the degradation of lignin, which typically occurs over a broader range from 200°C to 600°C. The DSC graph in figure 5c indicates that the melting temperature of neat HDPE, approximately 137°C, decreased to a range of 132°C–134°C with the incorporation of PKS particles. This reduction in the melting temperature did not show a significant dependence on the ratio of the mixed particle sizes.

## 4. Conclusion

The present study investigated the synergistic effects of incorporating mixed-filler sizes of PKSs into HDPE on the tensile properties of PKS/HDPE composites. The results demonstrated that smaller-sized PKS particles generally enhanced the tensile properties of the composites, including the UTS, Elastic Modulus (E), Total Elongation (εF), Yield Strength (σY), and Stress at Failure (σF).

Conversely, incorporation of larger PKS particles significantly diminished the tensile properties of the composites. This decline in performance became more pronounced with increasing concentrations of the larger particles. SEM analyses revealed that debonding was a critical factor contributing to the failure of the composites, particularly at higher concentrations of larger-sized particles (e.g. 7.5 wt% PKSL, 15 wt% PKSL and 22.5 wt% PKSL). This observation suggests inadequate interactions between the filler–filler and filler–matrix interfaces, leading to a compromised mechanical integrity.

Future research should focus on exploring the effects of combining PKS particles of various sizes, including both larger and smaller ones, to better understand the overall impact on the tensile properties of the composite. Additional mechanical testing, such as impact and hardness assessments, is recommended to identify the specific mechanical properties that are most enhanced by the composite. Such investigations will aid in determining potential applications of these composites. Furthermore, the surface treatment of larger-sized PKS particles can be explored to improve the interfacial bonding between the matrix and filler. Enhanced bonding can mitigate some of the performance issues associated with larger particles and improve the overall effectiveness of the composite material.

## Data Availability

The data used for this study can be accessed through Dryad [33].
